# Structural Brain Network Characteristics Can Differentiate CIS from Early RRMS

**DOI:** 10.3389/fnins.2016.00014

**Published:** 2016-02-02

**Authors:** Muthuraman Muthuraman, Vinzenz Fleischer, Pierre Kolber, Felix Luessi, Frauke Zipp, Sergiu Groppa

**Affiliations:** Department of Neurology and Neuroimaging Center of the Focus Program Translational Neuroscience, University Medical Center of the Johannes Gutenberg-University MainzMainz, Germany

**Keywords:** multiple sclerosis, diffusion tensor imaging, cortical thickness, connectivity, support vector machines

## Abstract

Focal demyelinated lesions, diffuse white matter (WM) damage, and gray matter (GM) atrophy influence directly the disease progression in patients with multiple sclerosis. The aim of this study was to identify specific characteristics of GM and WM structural networks in subjects with clinically isolated syndrome (CIS) in comparison to patients with early relapsing-remitting multiple sclerosis (RRMS). Twenty patients with CIS, 33 with RRMS, and 40 healthy subjects were investigated using 3 T-MRI. Diffusion tensor imaging was applied, together with probabilistic tractography and fractional anisotropy (FA) maps for WM and cortical thickness correlation analysis for GM, to determine the structural connectivity patterns. A network topology analysis with the aid of graph theoretical approaches was used to characterize the network at different community levels (modularity, clustering coefficient, global, and local efficiencies). Finally, we applied support vector machines (SVM) to automatically discriminate the two groups. In comparison to CIS subjects, patients with RRMS were found to have increased modular connectivity and higher local clustering, highlighting increased local processing in both GM and WM. Both groups presented increased modularity and clustering coefficients in comparison to healthy controls. SVM algorithms achieved 97% accuracy using the clustering coefficient as classifier derived from GM and 65% using WM from probabilistic tractography and 67% from modularity of FA maps to differentiate between CIS and RRMS patients. We demonstrate a clear increase of modular and local connectivity in patients with early RRMS in comparison to CIS and healthy subjects. Based only on a single anatomic scan and without a priori information, we developed an automated and investigator-independent paradigm that can accurately discriminate between patients with these clinically similar disease entities, and could thus complement the current dissemination-in-time criteria for clinical diagnosis.

## Introduction

Multiple sclerosis is a neuroinflammatory disease of the central nervous system leading to progressive disability in young adults (Noseworthy et al., 2000; Confavreux and Vukusic, 2006; Alonso and Hernán, 2008). The underlying pathology of multiple sclerosis is characterized by inflammation, demyelination, axonal injury, and axonal loss (Siffrin et al., 2010; Zipp et al., 2013), leading to a disruption of short- and long-range connections that results in functional deficits. The simultaneous processes of damage and repair are hallmarks of the disease, although they cannot easily be quantified. Furthermore, the continuous structural reorganization that maintains network function plays an essential role in the long-term outcome (Tomassini et al., 2012). Particularly in the initial phase of the disease, an exact understanding of the structural changes that occur is crucial, since in this period, important therapeutic decisions could influence the subsequent disease course.

Between 38 and 68% of patients with a first sub-acute neurological event suggestive of multiple sclerosis go on to develop clinically definite relapsing-remitting multiple sclerosis (RRMS; Poser and Poser, 1983; Eriksson et al., 2003; Confavreux and Vukusic, 2006; Fisniku et al., 2008). The definite diagnosis of RRMS based on the current revised McDonald diagnostic criteria requires clinical or radiological evidence of dissemination in space (DIS) and dissemination in time (DIT) of lesions typical of multiple sclerosis (Swanton et al., 2007; Polman et al., 2011). Patients who do not fulfill these diagnostic criteria after one clinical episode (mostly due to absence of DIT), but whose symptoms are still suggestive of multiple sclerosis, are classified as having clinically-isolated syndrome (CIS; Miller et al., 2005, 2012). Patients with CIS can convert to the relapsing-remitting course of multiple sclerosis depending on clinical or MRI markers (Moraal et al., 2009). From the clinical perspective, a rapid transformation from CIS to RRMS is associated with clear progression and a poor long-term outcome (Weinshenker et al., 1989; Achiron and Barak, 2000). From the radiological point of view, it has been shown that T2 lesion load can predict the conversion to definite multiple sclerosis (Filippi et al., 1995; Gauthier et al., 2007). Moreover, several recent studies have concentrated on gray matter changes and brain atrophy as promising markers to track the conversion from CIS to RRMS (Calabrese et al., 2011; Uher et al., 2014). Notably, patients experiencing a rapid transformation within 1 year show cortical atrophy in contrast to patients with slower disease progression (Pérez-Miralles et al., 2013).

Despite advances in clinical and MRI markers of conversion and disability progression, conventional MR abnormalities found in the initial scan often cannot unequivocally distinguish CIS patients from those with RRMS. This means that the combination of clinical and radiological evidence must still be relied upon to reach a diagnosis, potentially delaying it and affecting important therapeutic decisions.

Our study aims to identify MRI-derived hallmarks to distinguish between CIS and RRMS patients. We postulate that gray or white matter network characteristics can differentiate between CIS and RRMS based on only the initial scan. To confirm this, we investigated white matter networks using DTI and probabilistic tractography (Groppa et al., 2014) and gray matter networks using cortical thickness measurements (Hanganu et al., 2015). Focusing on network changes and not on local structural properties might represent an important advancement toward a more accurate characterization of the clinical course. By providing a complete morphometric outline of the brain (considering both white and gray matter tissue compartments), we were able to track the connectivity patterns as a possible surrogate marker of early disease progression, at which time no visible structural abnormalities could be detected between CIS and RRMS. Finally, applying a support vector machine classifier (Chang and Lin, 2011), we propose an automated algorithm to distinguish the two disease forms independently of clinical observations and the investigator based on a single MRI scan.

## Methods

### Patient characteristics

Patients were recruited at the Neurology Department of the Medical Centre of the Johannes Gutenberg-University of Mainz. Twenty patients with CIS (mean age 33.8 ± 9.53 years; mean disease duration, 1.95 ± 1.79 months) and 33 RRMS patients (mean age, 32.7 ± 10.3 years; mean disease duration, 1.51 ± 1.73 months) were included in this study. We also enrolled a group of 40 age-matched healthy controls (mean age, 32.2 ± 9.1 years). The RRMS patients fulfilled the revised McDonald diagnostic criteria (Polman et al., 2011). Patients with CIS had MRI lesions typical of multiple sclerosis and a single clinical presentation suggestive of multiple sclerosis without fulfilling the McDonald DIT diagnostic criterion. Each patient was assessed clinically by an experienced neurologist to determine the Expanded Disability Status Scale (EDSS) score. At the time of the MR scan, 32 (60.4%) patients (19 [57.8%] RRMS, and 13 [65.0%] CIS) were on immunomodulatory treatment.

### Structural and diffusion-tensor MRI

MRI was performed on a 3 Tesla magnetic resonance scanner (Magnetom Tim Trio, Siemens Healthcare, Erlangen, Germany) with a 32-channel receive-only head coil at the Neuroimaging Center (NIC) Mainz, Germany with a standardized MS protocol (Droby et al., 2015).

Imaging was performed using sagittal 3D T1-weighted magnetization prepared rapid gradient echo (MP-RAGE) sequence (TE/TI/TR = 2.52/900/1900 ms, flip angle = 9°, field of view (FOV) = 256 × 256 mm^2^, matrix size = 256 × 256, slab thickness = 192 mm, voxel size = 1 × 1 × 1 mm^3^), sagittal 3D T2-weighted fluid attenuated inversion recovery (FLAIR) sequence [TE/TI/TR = 388/1800/5000 ms, echo-train length = 848, field of view (FOV) = 256 × 256 mm^2^, matrix size = 256 × 256, slab thickness = 192 mm, voxel size = 1 × 1 × 1 mm^3^], and diffusion tensor imaging (DTI) sequence with single-shot echo-planar read-out (TE/TR = 102/9000 ms, *b* = 0.900 s/mm^2^, 30 directions, one average, field of view (FOV) = 256 × 256 mm^2^, matrix size = 128 × 128, 62 slices, slice thickness = 2.5 mm, voxel size = 2 × 2 × 2.5 mm^3^). Major anatomical abnormalities were excluded by a neuroradiologist based on the subject's T1-weighted and FLAIR images of the whole brain.

### DTI analysis

**Figure 1:**
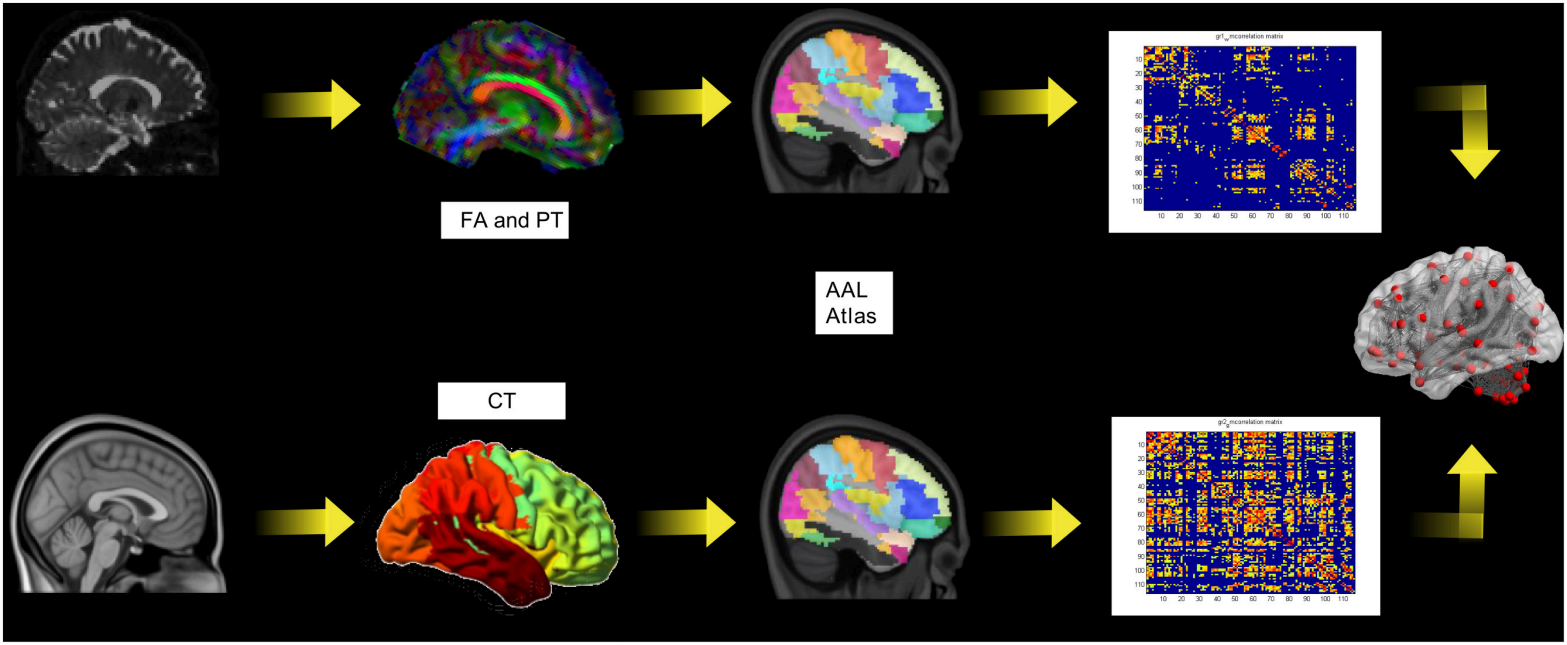
**The pipeline for the diffusion tensor imaging analysis including probabilistic tractography (PT) and fractional anisotropy (FA; upper row) and the cortical thickness (lower row)**.

The whole analyses pipeline is shown in Figure 1 (upper row). The image data was converted to the NIfTI format (dcm2nii, http://www.mccauslandcenter.sc.edu/mricro/) and processed using FSL (http://www.fmrib.ox.ac.uk/fsl/). A detailed protocol can be found elsewhere (Smith et al., 2006). In the following we give only a short description of the aspects relevant to this study. For the eddy current correction and further analysis we used the diffusion toolbox (FDT, part of FSL) and calculated the quantitative measures of fractional anisotropy (FA). Crossing fiber distribution was estimated using BEDPOSTX (implemented in FSL) and the probability of major and secondary fiber directions were calculated (Behrens et al., 2007). All images were aligned and affine-transformed into the stereotactic space MNI-152.

### Probabilistic tractography and white matter network reconstruction

To allow bias-free definition of seed and target areas unaffected by subjective judgments of anatomical correspondences, we constructed seed masks for 116 regions defined by the Automated Anatomical Labelling (AAL) atlas (Tzourio-Mazoyer et al., 2002). The aim of our tractography analysis was to generate ROI-based connectivity index maps of the entire brain diffusion network. A multi-fiber model was fit to the diffusion data at each voxel, allowing for tracing of fibs through regions of crossing or complexity (Behrens et al., 2003). Here, we drew 5000 streamlines from our seed voxels to form an estimate of the probability distribution of connections from each seed voxel within the AAL ROI. The probabilistic tractography connectivity matrix *PT*_*ij*_ (size 116 × 116) was generated for each subject, wherein each cell represents the number of samples (or streamlines) that passed through that ROI (*j*) to all generated streamlines from (*i*). These connectivity matrices were further included in the network analysis (Section Network Analysis below). FA maps were extracted from each AAL ROI. This data was used for the construction of the structural correlation matrices *S*_*ij*_ *S*_*ij*_ (size 116 × 116) for each group, in which each element represented the Pearson correlation coefficient between ROI values across subjects in one group.

### Cortical thickness and gray matter network reconstruction

The whole analyses pipeline is shown in Figure 1 (lower row). The construction of the cortical surface was based on 3D-T1 images using Freesurfer version 5.3.0 (http://surfer.nmr.mgh.harvard.edu). The detailed procedure for surface reconstruction has been described and validated in a previous study (Hanganu et al., 2015). For connectivity matrix reconstruction we applied the same node definition as above. We extracted the individual cortical thickness values for each ROI from the AAL-Atlas. This data was used as above described for FA values for the construction of the structural correlation matrices.

### Network analysis

Finally, the connectivity matrices were incorporated into the network analysis to describe the topological organization of structural cortical networks in our patients. In this study, we adopted two different analysis strategies in order to appropriately quantify the network topologies.

- For the probabilistic tractography (*PT*_*ij*_) matrices, we calculated the network measures in every subject and included this data in the further network topological parameter analysis.
- Cortical thickness and FA (*S*_*ij*_) connectivity matrices were calculated for each group. Afterwards the network topological parameters were calculated for the group.

In order to assess the network connectivity at different levels, we analyzed the following measures of network topology.

#### Modularity

Brain networks can be separated into modules. Modules are densely interconnected nodes that have only sparse interconnections to other modules. Modularity is defined as the relation of the within-module and between-module connections (Girvan and Newman, 2002; Newman, 2006b). In this study, the modularity (Q) was calculated with the Newman algorithm (Newman, 2006a,b),

$$\begin{array}{l} Q=\frac{1}{2m}\underset{ij}{\sum }\left[A_{ij}-P_{ij}\right]\;\delta \;\left(g_{i},g_{j}\right), \end{array}$$

where *A*_*ij*_ is the actual number of edges falling between a particular pair of vertices *i* and *j*; *P*_*ij*_ is the probability for an edge to fall between every pair of vertices *i*, *j*; *g*_*i*_ is the community to which vertex *i* belongs; *m* is the number of edges in the network; and δ(*r, s*) = 1 if *r* = *s* and 0 otherwise (Newman, 2006a).

#### Clustering coefficient

The clustering coefficient is a parameter of the local organization that reflects the number of connections between the neighbour's nodes (Watts and Strogatz, 1998). Highly interconnected neighbors form a cluster around the node, while sparsely interconnected neighbors do not. For our study, the clustering coefficient values have been calculated by the algorithm of Onnela et al. (2005),

$$\begin{array}{l} C_{i}=\frac{2t_{i}}{k_{i}\left(k_{i}-1\right)}, \end{array}$$

where *k*_*i*_ is the degree of the node *i* and *t*_*i*_ is the number of triangles attached to the node.

#### Local efficiency

While modularity and clustering coefficient describe the local network connectivity and segregation, it is essential to quantify the local integration or the efficiency of information flow. Local efficiency measurements have been introduced as the average of the inverse distance matrix in a sub-cluster (Latora and Marchiori, 2001). A fully connected network has maximal local efficiency since all distances are equal to one, while a disconnected network has minimal local efficiency since all distances are infinite. In this work we calculated the local efficiency with the Dijkstra's algorithm (see below; Latora and Marchiori, 2001; Onnela et al., 2005).

#### Global efficiency

Not only local network properties are essential for the network performance, but a further quantification of the system capacity to perform more global tasks, which go beyond the boundaries of cluster or modules, is needed. Global efficiency has been introduced as an entire network integration parameter to describe the information flow in the whole network. Similarly to the local efficiency, the global efficiency is an average of the inverse of the distance matrix but of the entire network matrix (Latora and Marchiori, 2001). Dijkstra's algorithm has been applied in our study to quantify the global efficiency (Latora and Marchiori, 2001; Onnela et al., 2005).

$$\begin{array}{l} E_{glob}\left(G_{i}\right)=\frac{1}{N\left(N-1\right)}\underset{i\neq j\in G}{\sum }\frac{1}{d_{ij}} \end{array}$$

where *E*_*glob*_(*G*_*i*_) is the global efficiency of *G*_*i*_, the subgraph of the neighbors of node *i*; and *d*_*ij*_ is the shortest path length between node *i* and node *j* in *G*.

$$\begin{array}{l} E_{loc}\left(G\right)=\frac{1}{N}\underset{i\in G}{\sum }E_{glob}\left(G_{i}\right) \end{array}$$

where *E*_*loc*_(*G*_*i*_) is the local efficiency of *G*, and *N*is the set of all nodes in the network.

For the above described network parameters, 20 density intervals (range 0.1–0.6) were calculated and compared between the two groups.

### Support vector machine (SVM) classification

SVM is a powerful tool (Cortes and Vapnik, 1995) for non-linear classification between two data sets. In short, the algorithm looks for an optimally separating threshold between the two data sets by maximizing the margin between classes' closest points. The points lying on the boundaries are called support vectors, and the middle of the margin is the optimal separating threshold. In most cases the linear separator is not ideal so a projection into a higher-dimensional space is performed where the data points effectively become linearly separable. Here, we have used the polynomial function kernel for this projection due to its good performance as discussed in Cortes and Vapnik (1995) and use the grid search (min = 1; max = 10) to find the few optimal input parameters namely C (Type of classification algorithm; 1 to 1000) and gamma (0.25). The selection was checked by 10-fold cross validation by taking 75% of the data for training and 25% for testing. A soft-margin classifier of the calculated network topology measurements was used for every parameter, and misclassifications were weighted by a penalty constant C. In order to optimize classification accuracy this was calculated for every classifier. The validation scheme was used to assess whether the included parameters of network topology allow automated classification between groups. The vectors from the connectivity metrics modularity, clustering coefficient, local and global efficiency over the analyzed densities were extracted and tested for the optimal parameter. The classification was conducted separately for each analyzed parameter of white and gray matter network topology. In order to have more robust testing phase we repeated the analyses for gray matter clustering coefficient and white matter modularity for three different testing data samples for the SVM. In order to demonstrate that no over fitting is attested in our data for the SVM classification algorithm we performed following steps. The results from the SVM were reported with 10-fold cross validation. Additionally, we estimated the receiver operating characteristics curve (ROC) for the four parameters global efficiency, local efficiency, global efficiency, and clustering coefficient separately.

### Statistical analysis

Statistical analysis of clinical data was performed using SPSS Statistics, Version 22.0 (SPSS, Chicago, Illinois, USA). Means and standard deviations (SD) as well as medians with ranges were calculated. A non-parametric Mann–Whitney test was performed to assess significant differences on the demographic data between the two groups.

The network comparison and the regional network analysis were estimated with a 95% confidence interval to extract the differences between the two groups of patients. The network parameters were compared with a two-tailed *t*-test at *p* < 0.05. We included several links densities to adjust the network topology measurements. Link density was defined as the ratio between the number of links in the network and the maximum possible number of links. The calculated topology parameters were tested to show the specificity of the effects to network reorganization and not differences due to a different total number of links.

## Results

Clinical variables such as age at MRI, age at diagnosis and gender, as well as disease duration and EDSS score did not significantly differ between the two patient groups (Table 1). Volumetric measurements including total brain volume, gray and white matter volumes and lesion volume also revealed no significant difference.

**Table 1:** **Demographic data and MR volume measurements for the CIS and RRMS patient groups**.

**Demographics**	**CIS Group (*n* = 20)**	**RRMS Group (*n* = 33)**	***p*-value**
Gender (male/female)	9/11	6/27	0.0519
Mean age (years)	33.85 (9.53)	32.67 (10.3)	0.6713
Mean age at diagnosis (years)	33.65 (9.60)	32.5 (10.4)	0.6853
Mean disease duration (months)	1.95 (1.79)	1.51 (1.73)	0.3861
Median EDSS (range)	1.1 (0–3.0)	1.45 (0–3.5)	0.2252
**VOLUMETRIC ANALYSIS**			
Mean WM volume (ml)	591.4 (68.3)	561.2 (73.9)	0.1437
Mean GM volume (ml)	635.4 (60.5)	630.7 (60.0)	0.7795
Mean TB volume (ml)	1445.58 (110.7)	1406.3 (129.1)	0.1795
Mean Lesion volume (ml)	2.878 (4.46)	5.4 (8.5)	0.2305

The standard deviation for all the parameters is given in brackets.

### White matter: probabilistic tractography

In order to differentiate the group network architecture patterns at the local level we computed clustering coefficient (*C*_*i*_) and local efficiency (*E*_*loc*_). Modularity (*Q*) and global efficiency (*E*_*glob*_) have been calculated as measures of the community and global connectivity. The modularity was significantly higher (*t* = 2.03; *p* = 0.03) over eight density intervals in RRMS patients compared to CIS patients. Furthermore, the clustering coefficient was higher in RRMS than in CIS. In the case of the clustering coefficient, seven density intervals were significantly higher (*t* = 2.13; *p* = 0.02) for the RRMS patients. In addition, global efficiency was higher in CIS than RRMS, with 12 density intervals significantly higher in CIS (*t* = 2.98; *p* = 0.001). Finally, the local efficiency was higher in CIS than RRMS. The local efficiency at all 20 density intervals did not show a significant difference between the two cohorts (*t* = 0.53; *p* = *0.1*). The network visualization of regional clustering coefficients and modules for CIS and RRMS patients is shown in **Figure 3** (upper row). The main differences between CIS and RRMS in the clustering coefficients were seen in the superior parietal region on both sides, as can be seen in **Figure 4A**. No significant differences in the number of modules were found between CIS and RRMS groups. However, the number of regions in each module differed. The main differences were noted in the module in the parieto-occipital region, with an increased number of nodes in RRMS (*n* = 31) compared to CIS (*n* = 5). However, in modules encompassing mesio-temporal regions and the cerebellum, a decreased number of modules were found in RRMS patients relative to CIS (mesio-temporal region, *n* = 21 vs. *n* = 29; cerebellum, *n* = 26 vs. *n* = 41).

The group of healthy individuals exhibited lower modularity (*t* = 3.48; *p* < 0.001, *t* = 3.65; *p* < 0.001) and clustering coefficient (*t* = 3.52; *p* < 0.001, *t* = 3.21; *p* < 0.001) values than CIS and early RRMS patients. In the case of global efficiency, the healthy subjects showed significantly lower values (*t* = 3.46; *p* < 0.001) than the CIS patients. However, the RRMS showed higher values than the healthy subjects, although this difference was not significant (*t* = 2.06; *p* > 0.1) for white matter.

### White matter: fractional anisotropy maps

No significant differences were attested between the groups for the studied network architecture parameters. No significant differences in the number of modules were found between CIS and RRMS groups. However, the number of regions in each module differed. The main differences were noted in the module in the parieto-occipital region, with an increased number of nodes in RRMS (*n* = 32) compared to CIS (*n* = 15). However, in modules encompassing mesio-temporal regions and the cerebellum, a decreased number of modules were found in RRMS patients relative to CIS (mesio-temporal region, *n* = 23 vs. *n* = 27; cerebellum, *n* = 24 vs. *n* = 38).

The group of healthy individuals exhibited lower modularity (*t* = 3.56; *p* < 0.001, *t* = 3.71; *p* < 0.001) and clustering coefficient (*t* = 3.63; *p* < 0.001, *t* = 3.56; *p* < 0.001) values than CIS and early RRMS patients. In the case of global efficiency, the healthy subjects showed significantly lower values (*t* = 3.58; *p* < 0.001) than the CIS patients. However, the RRMS showed higher values than the healthy subjects, although this difference was not significant (*t* = 2.10; *p* > 0.1).

### Gray matter: cortical thickness

**Figure 2:**
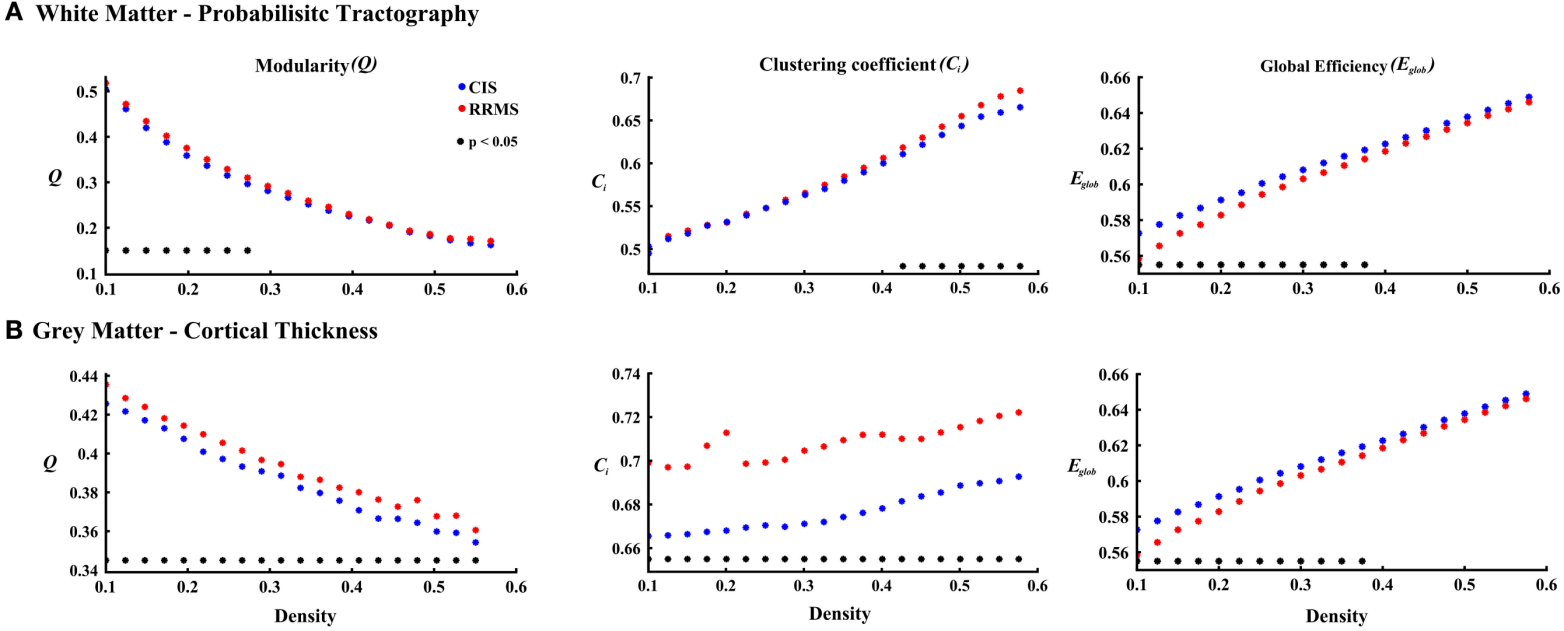
**The plots show the estimated values for the parameters modularity, clustering coefficient, and global efficiency for white matter (A) and cortical thickness (B) analyses**. The red asterisks indicate the RRMS patients and the blue asterisks represent the CIS patients. The black asterisks in each plot show the density intervals in which there was significant difference (*p* < 0.05) between the two groups.

**Figure 3:**
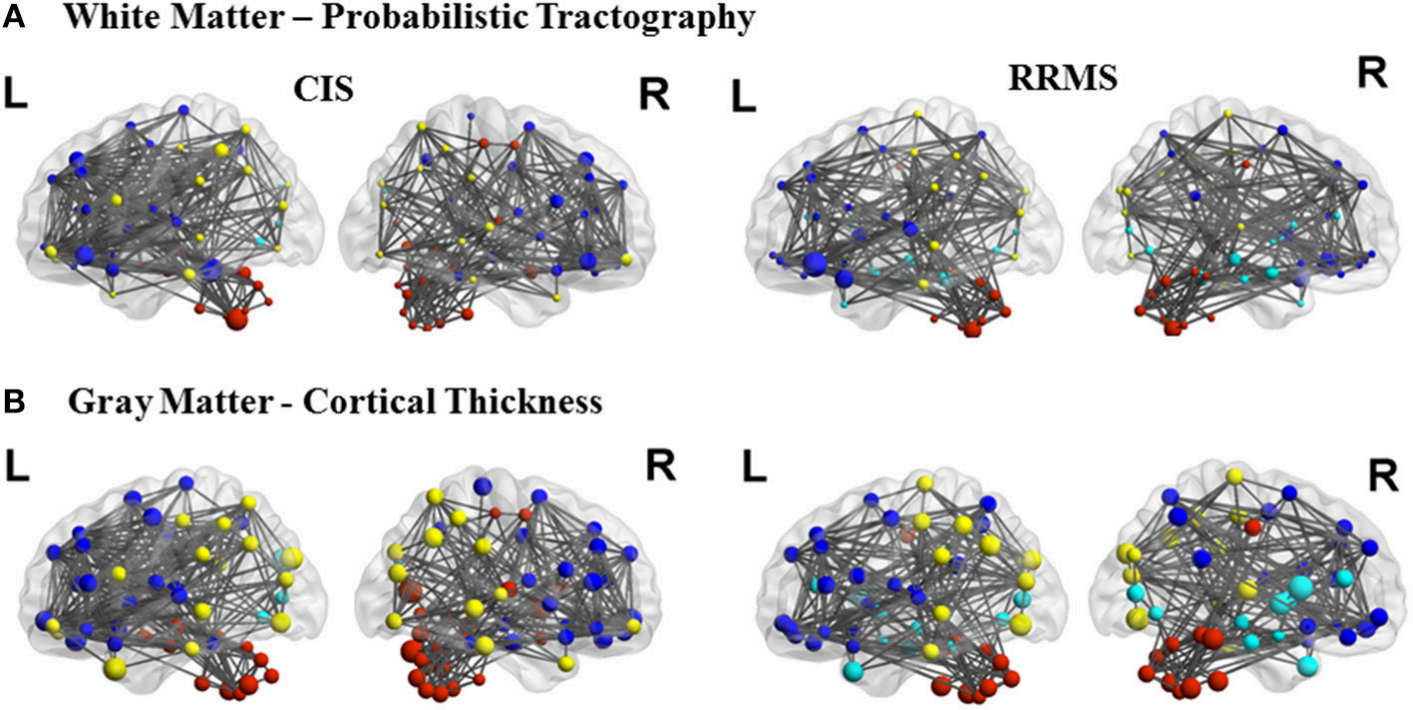
**Topological representation of the parameter clustering coefficient (size of the nodes), structural connections (links), and modules (color of the nodes) for CIS and RRMS**. Left (L) and right (R) hemispheres are shown. The first row shows the white matter connectivity derived from DTI and probabilistic tractography and the second row from the cortical thickness correlation analysis.

**Figure 4:**
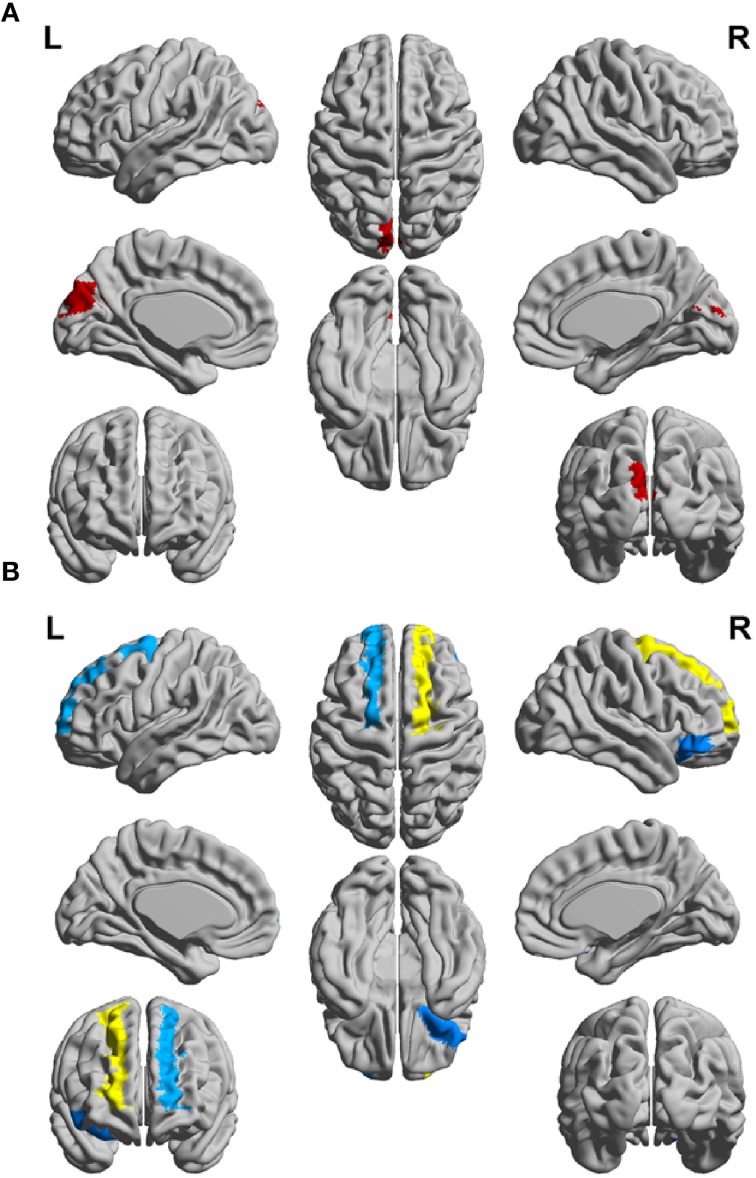
**Schematic representation of differences between the CIS and RRMS patient groups for clustering coefficient (CC) of (A) the white matter networks and (B) gray matter networks of the left (L) and right (R) hemispheres**.

Gray matter network architecture differed significantly between CIS and RRMS patients. Modularity was higher in RRMS compared to CIS (Figure 2), with values significantly increased over all 20 density intervals (*t* = 2.53; *p* = 0.006). The cluster connectivity pattern was notably increased in RRMS relative to CIS, with all 20 intervals showing significant differences (*t* = 3.41; *p* = 0.00001). However, global efficiency, as parameter of entire network connectivity, showed the opposite behavior, being significantly lower in RRMS patients than in CIS patients. For 12 density intervals the values significantly differed (*t* = 3.67; *p* = 0.00009). The local efficiency did not differ between the groups (*t* = 0.67; *p* = 0.2). Figure 3 (lower row) shows the network representation of the nodal clustering coefficients and module distribution. The main differences between CIS and RRMS in gray matter network representation of the clustering coefficients were seen bilaterally in the superior frontal gyrus (Figure 4B). Similarly to white matter, no significant differences in the number of modules could be determined between the CIS and RRMS groups. The number of nodes in each module was, however, different in CIS and RRMS patients, showing a higher number of nodes in the parieto-occipital module in RRMS (*n* = 35 vs. *n* = 10) but lower in the temporo-mesial (*n* = 23 vs. *n* = 31) and cerebellar (*n* = 22 vs. *n* = 37) regions in RRMS patients.

**Figure 5:**
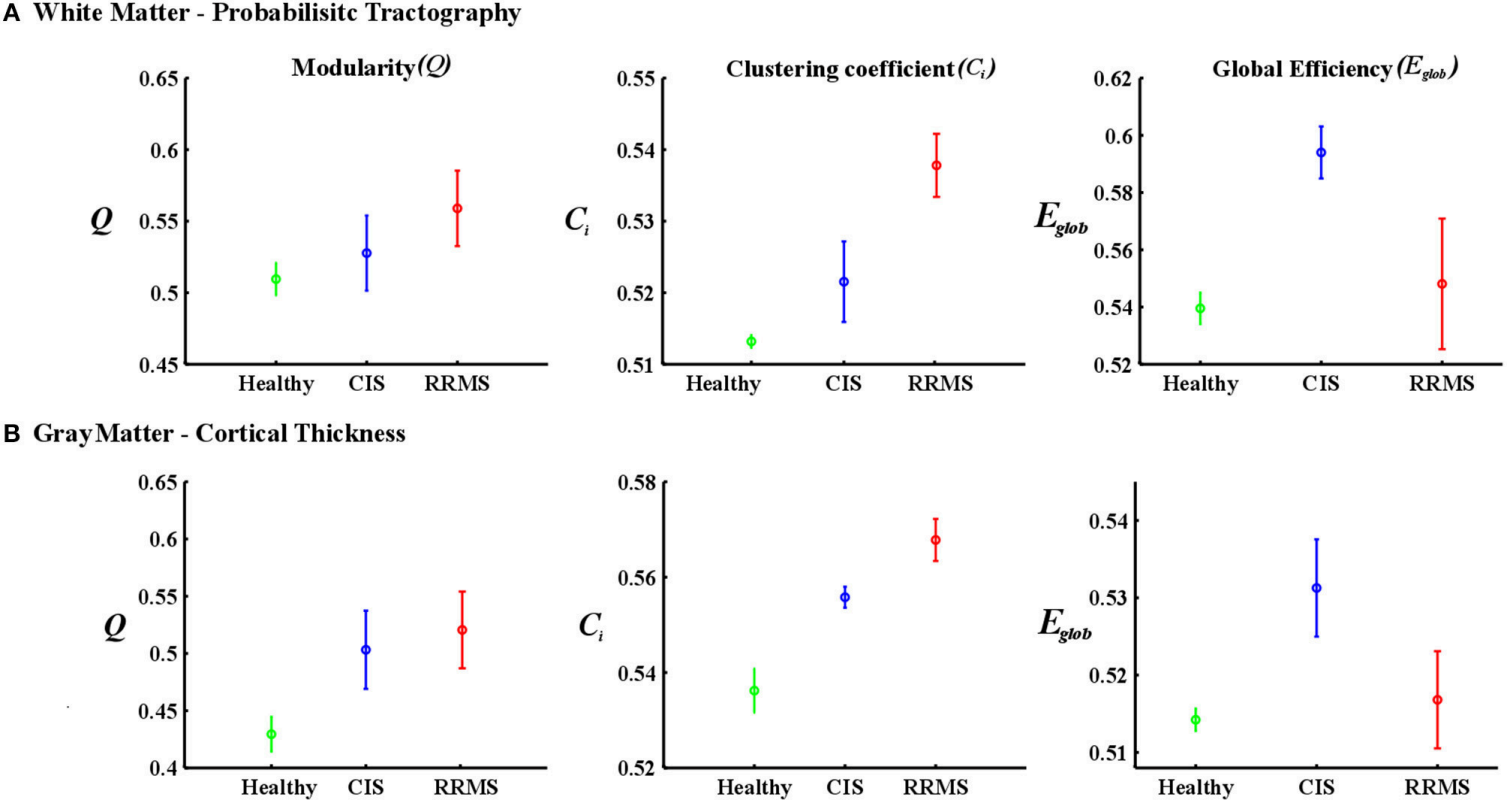
**Mean values and standard deviations over investigated densities**. **(A)** The first row is for the white matter-probabilistic tractography and **(B)** the second row is gray matter cortical thickness. The healthy group is represented in green, the CIS in blue and the RRMS in red.

In case of the gray matter, the group of healthy individuals showed lower modularity (*t* = 3.57; *p* < 0.001, *t* = 3.27; *p* < 0.001) and clustering coefficient (*t* = 3.76; *p* < 0.001, *t* = 3.84; *p* < 0.001) values than CIS and early RRMS patients. In the case of global efficiency, the healthy subjects showed significantly lower values (*t* = 3.04; *p* < 0.05) than the CIS patients. However, the RRMS showed higher values than the healthy subjects, although this difference was not significant (*t* = 2.12; *p* > 0.05). The results are depicted in Figure 5.

### SVM classification

**Figure 6:**
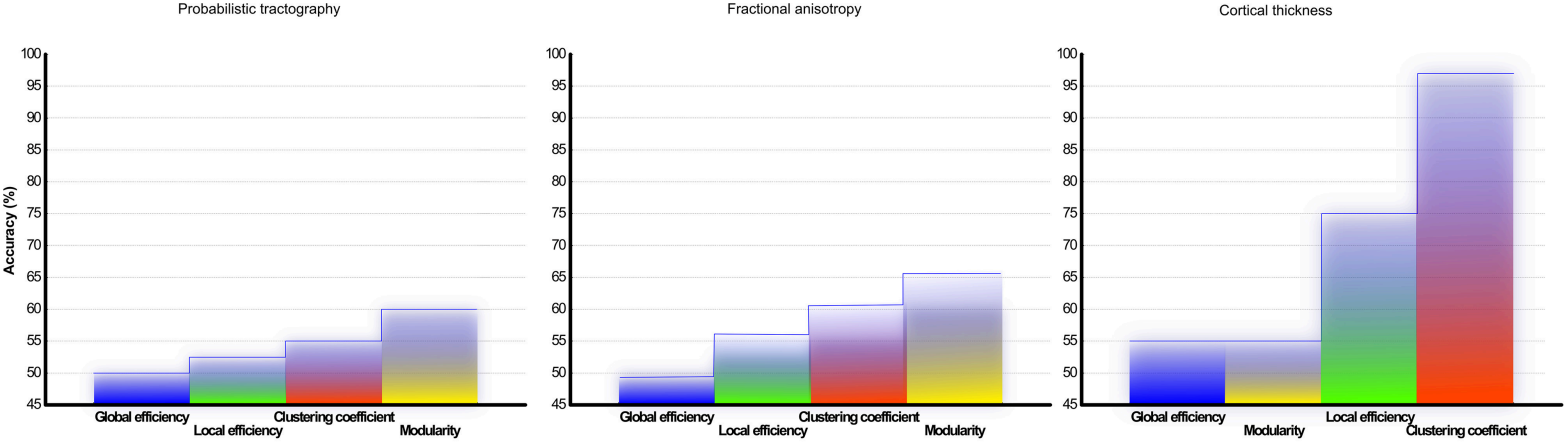
**Classification accuracy of CIS and RRMS: Results from the support vector machine analysis using classifier from network topology analyses measurements of (A) DTI and probabilistic tractography reconstruction of white matter and (B) fractional anisotropy (C) cortical thickness correlation matrix from gray matter**. The overall classification accuracy in (%) is shown.

To assess the predictive accuracy of network property values to distinguish between CIS and RRMS, we employed a SVM classifier as described in the Methods section. The classification accuracy for the analyzed four parameters using white and gray matter is depicted in Figure 6. In the case of white matter using PT, the maximal classification accuracy was obtained using modularity as the classifier, achieving 83.3% for training, 60.0% for testing, and 65% in the cross validation testing. For the white matter FA maps the maximum classification accuracy was obtained using modularity as the classifier, achieving 61% for training, 76% for testing, and 67% in the cross validation testing. Using the clustering coefficient derived from the gray matter cortical thickness, a maximum accuracy of 98% for training, 96% for testing, and 97% in the cross validation testing was achieved, highlighting the essential role of local gray matter architecture in differentiating the two disease entities. The SVM results were tested for 40 and 60% training datasets and the results are given in Table 2. The results from the receiver operating characteristics curve (ROC) for the four parameters global efficiency, local efficiency, global efficiency, and clustering coefficient separately for gray matter and the corresponding area under the curve (AUC), 95% confidence interval (CI), and *p*-values are given in Table 3.

**Table 2:** **SVM results for the three different training (%) data sets for parameters derived from cortical thickness**.

**Training data in % (Training/Testing)**	**Training Accuracy (%)**		**Testing Accuracy (%)**		**Overall Accuracy (%)**	
**Parameters**	***C*_*i*_**	**Q**	***C*_*i*_**	**Q**	***C*_*i*_**	**Q**
40(16/24)	88	77	89	71	89	60
60(24/16)	93	80	86	67	89	63
75(30/10)	98	83	96	60	97	65

**Table 3:** **The receiver operating curves (ROC) analyses results of the gray matter for the validation of no overfitting in this study**.

**Parameters**	**AUC**	**95% CI**	***p*-value**
Clustering Coefficient	1.00	1.00	<0.0001
Local Efficiency	0.54	0.36	0.32
Global Efficiency	0.56	0.38	0.25
Modularity	0.57	0.39	0.22

AUC, Area under the curve; 95% CI, confidence interval.

## Discussion

A diagnosis of RRMS is made when the dissemination in time and space criteria are fulfilled, and delaying diagnosis and treatment could detrimentally impact the long-term outcome. The goal of our study was to identify differences in the structural networks of CIS and RRMS patients following disease onset, and to determine clear objective morphologic criteria that differentiate between these groups. The technical aspect of paper is to find a feature which differentiates the two groups of patients in a streamlined and unconstrained way with a single anatomical MRI. We found patterns of higher local and modular processing in the gray matter of RRMS patients relative to those with CIS. The novelty of our study is the reconstruction of the entire white and gray matter structural connectome using probabilistic tractography and cortical thickness measurements. These methods possess tremendous advantages of providing an objective accurate description of microstructural integrity and surface topology that is closely linked to clinical anatomy and physiology. With the aid of SVM algorithms, we could differentiate patients with CIS from those with RRMS at a very high accuracy, merely on the basis of one structural MR scan and without a priori knowledge of clinical data or disease history.

Recent attempts have been made to analyse regional morphometric MRI differences in patients with CIS (Raz et al., 2010a,b; Cappellani et al., 2014; Uher et al., 2014) and RRMS (Chard et al., 2002; De Stefano et al., 2003; Debernard et al., 2014; Mallik et al., 2015); however, no clear group patterns have been described, and in most studies only comparisons to healthy controls were conducted. Studies on volumetric changes between CIS and RRMS are scarce and network analyses of CIS and RRMS patients in the very early period after disease onset have not yet been reported. It also remains unclear whether white or gray matter pathology dominates the early phase of the disease and how these structural markers reflect disease activity.

### White matter connectivity analysis

Several studies have focused on regional white matter changes in CIS (Cappellani et al., 2014; Uher et al., 2014) and RRMS (Chard et al., 2002; De Stefano et al., 2003), showing microstructural differences predominantly in comparison to healthy controls. In a cross sectional comparison with healthy controls, CIS patients presented a significant reduction in fractional anisotropy (FA), mainly in the corticospinal tracts, the corpus callosum, and the superior and inferior longitudinal fasciculi (Raz et al., 2010b). Similarly in RRMS, a reduction in FA in the thalamus, striatum, and corpus callosum was reported (Yu et al., 2012).

The main advantage of our approach is the consideration of the entire white matter structural network and not merely regional abnormalities. We found that RRMS patients exhibited a stronger modular structure in their white matter networks than patients with CIS, indicating that the nodes in the modules are more densely inter-connected in RRMS but exhibit fewer connections to other modules. In fact, this structural network reorganization pointing toward an increase in local processing and a decrease in long-range effectiveness is even recognizable in the comparison to healthy controls. Interestingly, this more distributed network structure is associated with increasing adaptability, as recently shown in studies of normal aging and neurodegenerative disorders (Kashtan and Alon, 2005). Such decomposability allows rapid adaptation and evolution in response to changing environmental conditions (Simon, 1995)—in this case to CNS inflammation. We postulate that the white matter networks in patients with definite RRMS undergo reorganization processes that either do not or have not yet occurred in those with CIS.

Besides increased modularity, we further detected a higher clustering coefficient in patients with RRMS, illustrating the strengthening of short distance connections and promoting of local integration in white matter networks. This “small world” property enables the whole network to work more efficiently compared to randomly structured networks. The main differences between CIS and RRMS in clustered, local processing (as shown in Figure 4) occurred in the white matter of the superior frontal lobe, showing its structural involvement in the early disease course. Furthermore, alterations of local connectivity were observed in the temporal lobe and cerebellum, which may be linked to the role of these structures in the compensation of network functioning as the disease progress (Rocca et al., 2012). These regions are also a part of the more global network that is increasingly activated in patients with CIS and RRMS in comparison to healthy controls when performing specific motor tasks as shown by fMRI data (Rocca et al., 2005; Dogonowski et al., 2014). One limitation in the network analysis is the fact that the reconstruction of the white matter network from the DTI data is based on probabilistic tractography. The diffusion-weighted MR signal provides useful qualitative results on the connection patterns of brain regions, however they are only the best possible guesses as the probability is rather an indication for increased connectivity (Jones et al., 2013).

### Gray matter connectivity analysis

A clear involvement of gray matter structures in early RRMS has been recently postulated (De Stefano et al., 2010). Gray matter atrophy and cortical lesions detected on double-inversion recovery images have also been recently associated with an increased risk of conversion to multiple sclerosis (Calabrese et al., 2011; Filippi and Rocca, 2012), and gray matter atrophy has been shown to be important for the long-term outcome and closely related with functional disability (Sepulcre et al., 2006; Fisniku et al., 2008), acting as a relevant predictor for disease progression (Bergsland et al., 2012). Indeed, gray matter atrophy correlated with disease subtype and disability progression to a greater extent than white matter changes or T2-hyperintensive lesions (Bonavita et al., 2011). Our data highlights clear morphometric changes involving gray matter very early in the disease, so focusing on changes in the gray matter to better understand and track the evolving pathology might help to advance clinical practice and research.

CIS patients do not show clear cortical gray matter atrophy in comparison to healthy subjects (Dalton et al., 2004; Calabrese et al., 2011); however, a discrete reduction of global cerebellar volume was noted. Patients who converted to RRMS during the follow-up developed a significant atrophy in the precentral gyrus and superior frontal gyrus, reflecting the described alterations of local connectivity observed in our study. We did not observe differences in the volumetric measurements of the analyzed groups, but the connectivity pattern did differ. The structural reorganization of gray matter networks in RRMS was manifest at the local and modular level, while at the entire network level, information processing decreased as shown by reduced global efficiency. Previously, it has been demonstrated that gray matter networks as analyzed by cortical thickness connectivity present a “small-world” architecture characterized by high clustering coefficients, and that this is continuously disrupted in RRMS patients with increasing lesion load (He et al., 2009), leading to rather inefficient cortical network processing. The emergence of a small-world topology in very early RRMS, as observed here, presumably ensures optimal information flow on the local level with “minimal costs” in response to inflammatory damage. Comparing white and gray matter networks at the global scale; our study reveals changes at the global network level with increased efficiency in gray matter and negligible alterations in white matter, suggesting separate reorganization patterns in these tissue types. The different structural evolution of white and gray matter global networks might be driven by functional compensation that is more strongly expressed in the gray matter.

### Group classification and clinical implications

Our approach has surpassed the classification accuracy based on solely clinical and manually derived lesion masks using an automated approach based on parameters from graph theoretical network connectivity matrices. We were able to differentiate CIS and RRMS patients using the clustering coefficient of the gray matter networks, and the algorithms applied were able to achieve a remarkable accuracy, indicating the pivotal role of early gray matter pathology in RRMS. These network changes occur and are measurable even prior to significant changes in brain volumes or gray matter atrophy. One limitation in this study is the number of patients which could be increased to larger sample size to check the robustness of this methodological pipeline for classification between CIS and RRMS patients. We were able to justify that no over fitting was there in our SVM classification results by the ROC curves which indicated the clustering coefficient from gray matter had the highest classification between the two groups of patients.

Using white matter network characteristics, we could achieve a maximum accuracy of 65% in group distinction. These clear and objective MR-driven group differences highlight the necessity of advanced analysing algorithms that mirrors the ongoing disease pathology to a better extend as conventional MR or MR-detectable lesions during the course of the disease This stresses that not only the classical white matter lesions, but possibly even more important eminent structural phenomena in the early disease course influence the disease progression. Indeed, analysing merely white matter lesions maps allows merely relatively low discrimination power (Weygandt et al., 2011). Furthermore, our developed SVM algorithms can independently discriminate similar clinical entities without reference to healthy controls who do no present lesions in the MRI. A recent study investigated whether machine learning techniques are able to predict the occurrence of a second attack in CIS patients based on MRI lesion distribution and clinical demographics (Wottschel et al., 2015). The accuracy of SVMs to correctly predict clinically definite RRMS assessed 1 year after the first clinical event was 71.4%, rather low compared to the higher accuracies achieved in other neurological diseases (Klöppel et al., 2008, 2009; Mwangi et al., 2012), highlighting how challenging it can be to properly classify these two groups of patients. Here, by considering differences in both gray and white matter network architectures and by making a direct comparison of patients without reference to healthy controls, we have achieved an accuracy of 97%. The robustness of the SVM results were tested for three different training sample size and the results were controlled for the overall accuracy for the clustering coefficient and modularity for gray and white matter respectively.

## Conclusion

In this study, we have demonstrated that CIS and RRMS patients differ in modular and local structural network architecture, most prevalently in gray matter but also measurable in white matter tissue. Based on this characterization, we identified network topology measurements that could be utilized in automated pattern recognition algorithms to discriminate between these groups with excellent precision. Our findings thus, provide evidence that MR-driven network analysis could serve as a diagnostic marker to more accurately track disease course and potentially make treatment decisions at an earlier disease stage.

## Author contributions

MM was involved in organization and execution of the research project; review and critique of the statistical analysis; writing of the first draft, review, and critique of the manuscript. VF and PK was involved in conception and organization of the research project; writing of the first draft, critique of the manuscript. FL was involved in organization and execution of the research project; critique of the manuscript. FZ was involved in organization and execution of the research project, writing, and critique of the manuscript. SG was involved in conception, organization, and execution of the research project; review and critique of the statistical analysis; review and critique of the manuscript.

### Conflict of interest statement

The authors declare that the research was conducted in the absence of any commercial or financial relationships that could be construed as a potential conflict of interest.
